# Effect of surface treatments on push-out bond strength of calcium silicate-based cements to fiber posts

**DOI:** 10.1186/s12903-021-01518-y

**Published:** 2021-03-19

**Authors:** Amr M. Elnaghy, Ayman Mandorah, Ali H. Hassan, Alaa Elshazli, Shaymaa Elsaka

**Affiliations:** 1 Department of Endodontics, Faculty of Dentistry, Mansoura University, Mansoura, 35516 Egypt; 2 Department of Restorative and Dental Materials, Faculty of Dentistry, Taif University P.O Box 11099, Taif, 21944 Saudi Arabia; 3 Department of Orthodontics, Faculty of Dentistry, King Abdulaziz University, Jeddah, Saudi Arabia; 4 Department of Restorative Dental Sciences, Vision Colleges, Jeddah, Saudi Arabia; 5 Department of Dental Biomaterials, Faculty of Dentistry, Mansoura University, Mansoura, Egypt

**Keywords:** Biodentine, Fiber post, Push-out bond strength, Surface treatments, Titanium tetrafluoride

## Abstract

**Background:**

To evaluate the effect of surface treatments on the push-out bond strength of Biodentine (BD) and white mineral trioxide aggregate (WMTA) to fiber posts.

**Methods:**

Two brands of fiber posts were used: Reblida post; RP and RelyX post; RX. Each type of post (n = 80/group) was divided into four groups (n = 20/group) and exposed to surface treatment as follows: Control (no treatment), sandblasting (SB), hydrofluoric acid (HF), and TiF_4_ 4 wt/v%. Each group was further subdivided into two subgroups (n = 10/subgroup) based on the type of CSCs used as follows: *Subgroup A:* BD and *Subgroup B:* WMTA. Push-out bond strength of BD and WMTA to glass fiber posts was assessed. Data were statistically analyzed using three-way ANOVA and Tukey’s test. A Weibull analysis was performed on the push-out bond strength data.

**Results:**

BD showed higher bond strength than WMTA (*P* < 0.001). The push-out bond strength for posts treated with TiF4 4 wt/v% showed greater bond strength than the other surface treatments (*P* < 0.05). The BD/RP-TiF_4_ 4 wt/v% showed the greater characteristic bond strength (σ_0_) (15.93) compared with the other groups. Surface treatments modified the surface topography of glass fiber posts.

**Conclusions:**

The BD/RP-TiF_4_ 4 wt/v% showed greater bond strength compared with the other groups. The TiF_4_ 4 wt/v% surface treatment enhanced the bond strength of BD and WMTA to glass fiber posts than the other treatments. Surface treatment of fiber post with TiF_4_ 4 wt/v% could be used to improve the bond strength with calcium silicate-based cements.

## Background

Calcium silicate-based cements (CSCs) revealed favorable clinical outcomes with different clinical applications [1–3]. Biodentine (BD; Septodont, Saint Maur des Faussés, France) and white mineral trioxide aggregate (WMTA) are CSCs that were used for different applications in endodontic treatment including pulp capping, repair of root perforations, pulpotomies, apical barrier, regenerative endodontics, and obturation of the entire root canal space [4–9]. MTA CSCs materials have certain limitations, including long setting time, discoloration of teeth, and difficulty in handling lead to the development of different CSCs to overcome these disadvantages [10–12].

One of the applications of CSCs is the treatment of non-vital immature permanent teeth [4]. This procedure is established by disinfection of root canal, placement of apical barrier together with root-filling materials [13]. The fracture resistance of simulated immature permanent teeth used BD with fiber post was compared with different canal filling materials [4]. It was suggested that BD combined with fiber post might reinforce the immature teeth with an apical BD plug [4]. Fiber posts improved the fracture resistance of the tooth because their flexural modulus mimic to that of dentin [4, 7, 14–17]. This biomimetic characteristic enhances the stress distribution which decreases the incidence of root fracture, the most critical form of failure [18–20].

Surface treatments had been suggested to improve the adhesion properties between bi-materials interface, by providing micromechanical and chemical retention between different constituents [21, 22]. Various surface treatments had been applied to enhance the bond strength between the fibre post and other restorative materials as composite core [22] and resin cements [19, 23] including sandblasting, silane coupling agent, and acid etching agents [19, 22–24].

The adhesion between the BD and fiber post was not evaluated. It is important to enhance bonding between BD and fiber post to reinforce the root canal as in cases of non-vital immature permanent teeth [4]. Consequently, the aim of this study was to assess different surface treatments on the push-out bond strength of BD and WMTA to different systems of glass fiber posts. The null hypotheses tested of were that: (1) Different surface treatments would not affect the adhesion between fiber post and CSCs materials; (2) Different type of posts would not present difference on adhesion regardless the surface treatments, and (3) Different CSCs materials would not affect the adhesion of the fiber post regardless the surface treatments.

## Methods

The sample size determination was performed for push-out bond strength test using GPower v3.1.3 software (University of Düsseldorf; Düsseldorf, Germany). A power analysis revealed that a sample size of 50 specimens per subgroup was found to meet the constraints of α = 0.05 and power = 0.95.

### Push-out bond strength

Two brands of glass fiber posts were used: Reblida post (RP; size # Ø 1.5, VOCO, Cuxhaven, Germany) and RelyX post (RX; size # 2, 3 M ESPE, St. Paul, MN, USA). Materials used in this study are presented in Table 1. Each type of post (n = 80) was divided into four groups (n = 20/group) and exposed to the surface treatment as follows:

- ***Group 1:*** control (C); no treatment.
- ***Group 2:*** sandblasting (SB); the specimens were treated with a tribochemical silica-coated (CoJet system; 3 M ESPE) with 30 µm aluminum oxide particles from a distance 10 mm at 2.5 bar pressure for 15 s.
- ***Group 3:*** hydrofluoric acid (HF); the specimens were treated with 9% HF (Ultradent Porcelain Etch, Ultradent Products, South Jordan, UT, USA) for 1 min [23, 25].
- ***Group 4:*** titanium tetrafluoride (TiF_4_); the specimens were immersed in TiF_4_ 4 wt/v% (Sigma Aldrich, Saint Louis, MO, USA) solution for 4 min [23].

**Table 1:** Materials that used in this study

Material	Product	Composition	Lot number	Manufacturer
Fiber post	Reblida post Size # Ø 1.5	70% glass fiber, 10% filler, 20% UDMA	1143115	VOCO, Cuxhaven, Germany
	RelyX post Size # 2	Glass fiber reinforced composite, methacrylate resin	173421109	3 M ESPE, St. Paul, MN, USA
Surface treatment	Sandblasting (CoJet system)	CoJet-Sand: Aluminum trioxide particles coated with silica, particles size: 30 µm	625642	3 M ESPE, St. Paul, MN, USA
	Hydrofluoric acid	Buffered 9% hydrofluoric acid	BCG4P	Ultradent Porcelain Etch, Ultradent Products, South Jordan, UT, USA
	Titanium tetrafluoride	Titanium tetrafluoride 4 wt/v%	MKBN0100V	Sigma Aldrich, Saint Louis, MO, USA
Calcium silicate-based cements	Biodentine	Powder: Tricalcium silicate, Dicalcium silicate, Calcium carbonate, Zirconium dioxide, Iron oxideLiquid: Calcium chloride, Hydrosoluble polymer	B18542A	Septodont, Saint Maur des Faussés, France
	White mineral trioxide aggregate	SiO_2_, K_2_O, Al_2_O_3_, Na_2_O, Fe_2_O_3_, SO_3_, CaO, Bi_2_O_3_, MgO. Insoluble residues of CaO, KSO_4_, NaSO_4_, and crystalline silica	45732	PD MTA White; Produits Dentaires SA, Vevey, Switzerland

The specimens that were treated with HF and TiF_4_ 4 wt/v% were ultrasonically cleaned in distilled water for 1 min and then air-dried. Each group was further subdivided into two subgroups (n = 10/subgroup) based on the type of CSCs used as follows:

- ***Subgroup A:*** BD (Septodont, Saint Maur des Faussés, France).
- ***Subgroup B: ***WMTA (PD MTA White; Produits Dentaires SA, Vevey, Switzerland).

A sticky wax was used to position the post perpendicularly on a square plastic plate. Then, the post was surrounded by a cylindrical plastic matrix (12 mm height × 10 mm diameter) [22, 24]. The cylinder was filled with the CSCs using a MAP system (MAP One, Produits Dentaires SA, Vevey, Switzerland). The specimens were stored at 37 °C and 100% humidity for 48 h. The specimens were exposed to 10,000 thermocycles in distilled water between 5 and 55 °C with 5-s transfer time and 30-s dwell time [4, 7, 26].

After that, the straight portion (10 mm) of each CSCs-post system was sectioned using a low-speed diamond saw (Isomet 1000, Beuhler Ltd., Lake Bluff, IL, USA) that resulted in 5 discs (n = 50 discs/subgroup). The push-out bond strength of each disc was tested using a universal testing machine (Model TT-B, Instron Co., Canton, MA, USA) and loaded with a cylindrical plunger (1 mm diameter) at 0.05 mm/min cross-head speed. The push-out bond strength (MPa) was calculated by dividing the load at failure (Newtons) by the bonding area (mm^2^). The bonding area was calculated using the following equation [22]:

$$A \, = \, 2r \, \times \, \pi \, \times \, h$$

where r is the post radius, h is the thickness of each post section, and π is the constant 3.14.

### Failure mode analysis

The debonded specimens were observed by stereomicroscope (ZEISS, Stemi 2000-C, Oberkochen, Germany) at 50 × for analyzing the failure pattern. Failure mode was classified as Type 1; adhesive failure between the CSCs and the post, Type 2; cohesive failure within the post, Type 3; cohesive failure within the CSCs, and Type 4; mixed failure [23]. The failure mode was evaluated by the same operator.

### Surface topography

A total of 20 specimens of each type of post (n = 5/group) were prepared and grouped as mentioned before. The specimens were sputter-coated with gold (Sputter Coater S 150A; Edwards, Crawley, England). A scanning electron microscope (JSM 5600 Lv JEOL, Tokyo, Japan) was used to characterize the surface topography of the specimens at magnifications of 500 × .

### Statistical analysis

Normality of data distribution was tested using Kolmogorov–Smirnov and the equality of variances with the Levene’s test. The data were normally distributed. Data of push-out bond strength were statistically analyzed (SPSS 22.0 software; IBM Corp., Armonk, NY, USA) using three-way ANOVA based on three factors (the type of post, type of treatment, and type of CSCs) and their interactions. Multiple comparisons were conducted by the Tukey’s test. The level of statistical significance was set at *P* < 0.05. A Weibull analysis (SuperSMITH software; Fulton Findings, Torrance, CA, USA) was performed on the push-out bond strength data.

## Results

**Fig. 1:**
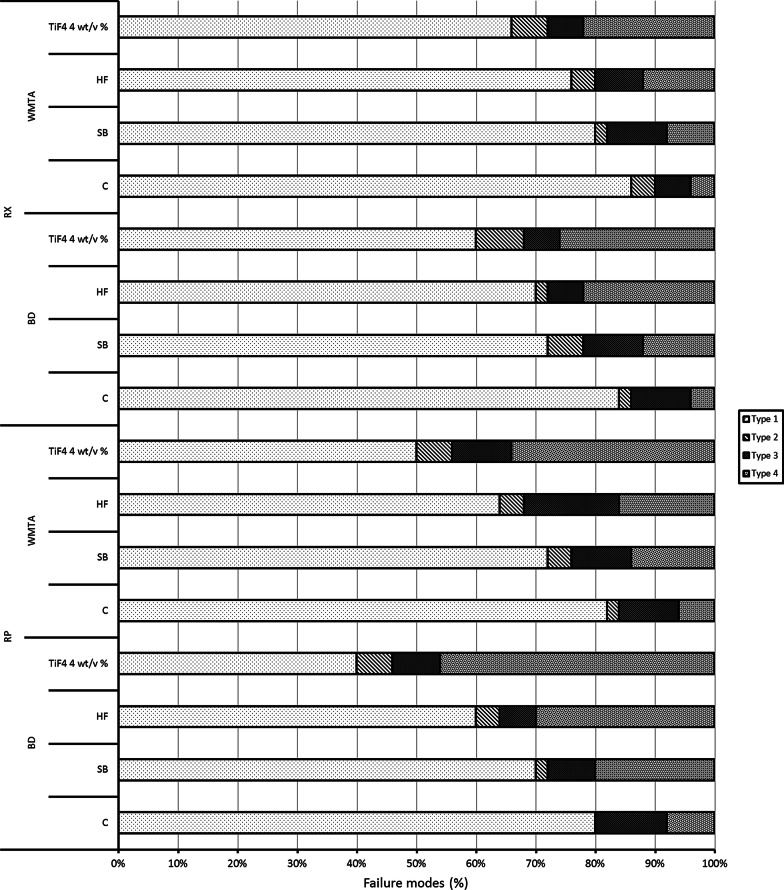
Failure modes distribution of different groups. Type 1; adhesive failure between the CSCs and the post, Type 2; cohesive failure within the post, Type 3; cohesive failure within the CSCs, and Type 4; mixed failure

Table 2 showed the results of 3-way ANOVA. The push-out bond strength was significantly influenced by the type of post, type of surface treatment, and type of CSCs (*P* < 0.001). The RP revealed higher bond strength to CSCs than RX (*P* < 0.001). Regarding the type of CSCs, the BD showed higher bond strength than WMTA (*P* < 0.001). There was no significant interaction between type of post and type of CSCs (*P* = 0.108) and between the type of CSCs and type of treatment (*P* = 0.394). There was a significant interaction between the type of post and type of treatment (*P* = 0.002). There was no significant interaction between type of post, type of CSCs, and type of treatment (*P* = 0.311). The mean and standard deviations of push-out bond strength (MPa) are presented in Table 3. The push-out bond strength for posts treated with TiF_4_ 4 wt/v% showed greater bond strength than the other surface treatments (*P* < 0.05). The improvement in the bond strength according to the surface treatments was as follows: TiF_4_ 4 wt/v% > HF > SB > C. Failure mode analysis of different groups is presented in Fig. 1. The higher percentage of failure modes was the adhesive failure between the post and CSCs (69.5%; Type 1). The other types of failure modes including mixed failures (17.7%; Type 4), cohesive failure within the CSCs (8.9%; Type 3), and cohesive failure within the post (3.9%; Type 2).

**Table 2:** Three-way ANOVA for the type of post, type of surface treatment, type of CSCs, and the interaction terms according to push-out bond strength data

Source of variation	Sum of squares	Df	Mean squares	F	*P *value
Type of post	136.951	1	136.951	166.987	< 0.001
Type of surface treatment	646.197	3	215.399	262.639	< 0.001
Type of CSCs	172.608	1	172.608	210.464	< 0.001
Post type × CSCs type	2.122	1	2.122	2.587	0.108
Post type × surface treatment	12.314	3	4.105	5.005	0.002
CSCs type × surface treatment	2.451	3	0.817	0.996	0.394
Post type × CSCs type × surface treatment	2.941	3	0.980	1.195	0.311
Total	13,290.960	800			

Statistically significant difference at *P* < 0.05

**Table 3:** Mean (standard deviation) values and statistical analysis of push-out bond strength (MPa) of different CSCs/post systems with different surface treatments

Surface treatment	CSCs/post systems			
	BD/RP	BD/RX	WMTA/RP	WMTA/RX
C	3.34 (0.78)^D^	2.73 (0.59)^D^	2.43 (0.59)^D^	2.13 (0.46)^D^
SB	4.38 (1.09)^C^	3.35 (1.01)^C^	3.12 (0.49)^C^	2.65 (0.73)^C^
HF	5.12 (1.12)^B^	4.13 (1.11)^B^	4.23 (0.95)^B^	3.19 (0.96)^B^
TiF_4_ 4 wt/v%	6.15 (1.03)^A^	5.05 (1.05)^A^	5.08 (1.05)^A^	3.99 (0.99)^A^

Mean values represented with different superscript uppercase letter (column) are significantly different (*P* < 0.05)

**Fig. 2:**
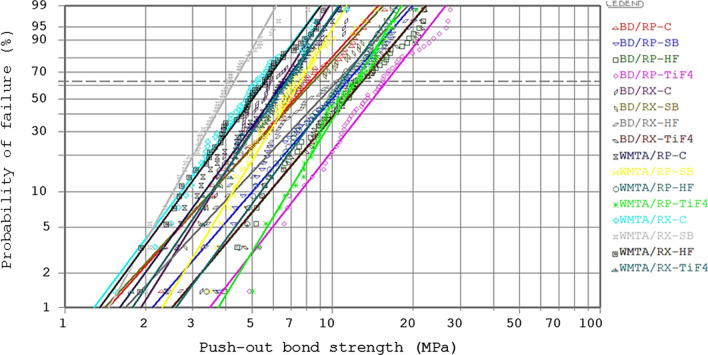
The Weibull plot of push-out bond strength (MPa) for different groups with different surface treatments. The dotted line is the characteristic strength. Surface treatments with TiF_4_ 4 wt/v% showed the highest characteristic bond strength compared with the other surface treatments. The BD/RP-TiF_4_ 4 wt/v% revealed the highest characteristic bond strength compared with the other groups

The data of Weibull analysis of different groups are presented in Table 4. The BD/RP-TiF_4_ 4 wt/v% showed the greater characteristic bond strength (σ_0_) (15.93) than the other groups. The surface treatment with TiF_4_ 4 wt/v% had more reliability than the other treatments (Table 4 and Fig. 2). The Weibull plot for different groups is presented in Fig. 2.

**Table 4:** Weibull analysis of push-out bond strength (MPa) of different CSCs/post systems with different surface treatments

Surface treatment	CSCs/post systems							
	BD/RP		BD/RX		WMTA/RP		WMTA/RX	
	Weibull modulus (m)	Characteristic bond strength (σ_0_)	Weibull modulus (m)	Characteristic bond strength (σ_0_)	Weibull modulus (m)	Characteristic bond strength (σ_0_)	Weibull modulus (m)	Characteristic bond strength (σ_0_)
C	2.63	8.31	3.78	6.5	3.24	6.65	3.15	5.54
SB	2.74	11.41	2.56	8.54	3.87	7.59	4.25	4.31
HF	2.8	12.9	2.53	10.42	3.22	10.86	3.22	5.64
TiF_4_ 4 wt/v%	3.02	15.93	2.81	12.91	3.89	12.23	3.46	6.77

**Fig. 3:**
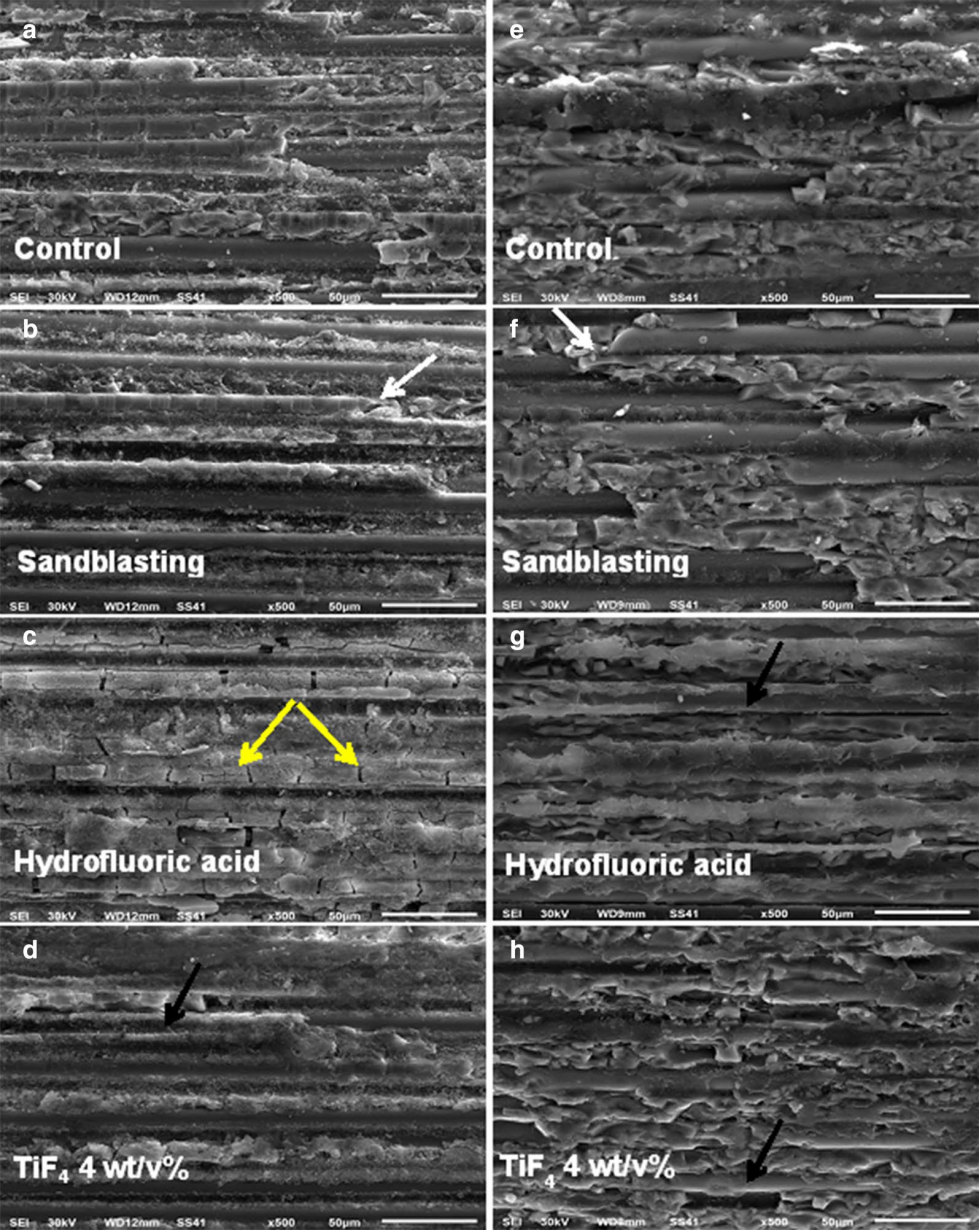
Representative SEM photomicrographs (×500) for the surface microstructure of **a**–**d** RP and **e**–**h** RX posts; respectively with different treatments. White arrows showed fractured glass fibers, yellow arrows showed the cracks in the glass fibers, and the black arrows showed the exposed glass fibers

Representative SEM photomicrographs for the surface microstructure of the different post systems with different treatments are shown in Fig. 3. The untreated RP showed exposed glass fibers with rather a rough surface than RX post that was typically more covered by the resin matrix (Fig. 3a, e; respectively). The sandblasted groups exhibited fractured glass fibers (Fig. 3b, f; respectively). For the RP post, the HF surface treatment caused cracks in the glass fiber (Fig. 3c). For the RX post, the glass fibers were exposed with HF surface treatment (Fig. 3g). The TiF_4_ 4 wt/v% surface treatments exposed the glass fibers of the RP and RX posts (Fig. 3d, h; respectively).

## Discussion

The present study evaluated the effect of surface treatment on bond strength of CSCs materials to fiber posts. The findings showed that the adhesion was considerably affected by the type of post, type of surface treatments, and type of CSCs. Accordingly, the null hypothesis was rejected.

It is significant to enhance bonding between CSCs and fiber posts to reinforce the root canals for treating non-vital immature permanent teeth [4]. In the present study, a cylindrical plastic matrix was used instead of a root specimen for testing the bond strength as the purpose of the study was to assess the bond strength between the posts and CSBs. There are limitations of using root dentin to test adhesion with fiber post including variation in hardness, elastic modulus, flexural strength, geometric parameters among different teeth that may affect the bond strength measurements [27, 28]. Consequently, specimens from the post and CSBs were prepared to test the adhesion between them without another factor that may affect the outcomes. All tested groups were subjected to thermocycling to simulate the clinical conditions that might provide a possible prediction of bonding durability [23]. It was postulated that approximately 10,000 thermal cycles correspond to 1 year of clinical function [26]. In the present study, TiF_4_ 4 wt/v% was used because it was shown in a previous study that this concentration enhanced the bond strength of resin cement to a fiber post [23]. Similarly, surface treatment of RP and RX posts with TiF_4_ 4 wt/v% for 4 min revealed greater bond strength to CSCs than the other treatments. This could be explained that TiF_4_ 4 wt/v% treatment might remove the surface layer of resin of the post which allows further areas for micromechanical retention with the CSCs [22, 23]. The TiF_4_ 4 wt/v% surface treatments exposed the glass fibers of the posts (Fig. 3d, h; respectively). In addition, surface treatments of fiber posts with TiF_4_ 4 wt/v% showed higher percentages of mixed failure modes (Type 4) than the other types of surface treatment.

The surface treatment with HF showed higher bond strength of CSCs to fiber posts than SB and C groups. The HF surface treatment might modify the outer surface layer of the fiber post without compromising the strength properties of the post [23, 25]. It was observed that HF surface treatment caused some cracks in RP post and the glass fibers were exposed in RX post (Fig. 3c, g; respectively). It was reported that HF surface treatment had a destructive effect on the surface integrity of the fiber post [29, 30].

Roughening the surface of fiber post with tribochemical silica coating might enhance the bond strength with the other bonded materials due to mechanical retention [23, 31]. Surface roughness with sandblasting increased the surface area of exposed glass fibers for bonding with the CSCs and accordingly enhancing the bond strength [23, 31]. Fiber posts that did not receive surface treatments showed the lowest bond strength with CSCs compared with the other groups. This finding indicates the importance of surface modifications of fiber posts to enhance the adhesion with CSCs.

The RP showed higher bond strength with CSCs than the RX post. This finding could be attributed to the different surface topography between RP and RX posts [22]. The surface topography of RP showed a relatively uneven surface with some uncovered glass fibers as shown in the control group (Fig. 3a) which provides more surface areas for mechanical retention with CSCs. The RP is composed of 70% glass fiber, 10% filler, and 20% urethane dimethacrylate [23]. However, the surface of the RX post was typically more enclosed by the resin matrix (Fig. 3e). The RX post is composed of glass fiber reinforced composite and methacrylate resin [22]. The differences in the composition of posts might influence their bond strength with the CSCs.

The BD revealed higher bond strength with RP and RX posts than WMTA. This enhancement in the bond strength might be due to the improved physical properties and the integrity of BD [5, 32] that might improve the bonding with the glass fiber posts. It had been reported that BD had a higher resistance to dislodgement than WMTA [32]. It could be postulated that BD had better mechanical adhesion with the posts after surface treatment that interlocks mechanically within the surface irregularities. CSCs materials should have adequate adaptation and a consistent adhesion to the post surface to reinforce the roots for treating non-vital immature permanent teeth [4].

The Weibull analysis was conducted on the push-out bond strength data. The BD/RP-TiF_4_ 4 wt/v% showed higher characteristic bond strength (σ_0_) than the other groups, comparable to the bond strength data. It was observed that TiF_4_ 4 wt/v% surface treatment had more reliability than the other treatments. It could be postulated that the clinical relevance of fiber post surface treatment with TiF_4_ 4 wt/v% might improve the bond strength with CSCs. One of the limitations of the present study is to mimic the clinical conditions in practice for restoring endodontic treated teeth with CSBs and glass fiber post. In addition, other parameters existing in the oral environment such as fatigue loading, temperature changes, and constant moisture could influence the performance of glass fiber post and CSBs. Due to the limitations of in vitro studies, further clinical studies are essential to assess the performance of treated glass fiber posts with the CSCs to restore endodontic treated teeth to give reliable recommendations for dental practitioners.

## Conclusions

BD revealed higher bond strength to fiber posts than WMTA. The TiF_4_ 4 wt/v% surface treatment enhanced the bond strength of BD and WMTA to glass fiber posts than the other treatments. The RP post improved the bond strength to CSCs than RX posts.

## Data Availability

The datasets generated and analyzed during the current study are not publicly available due to (ownership of data) but are available from the corresponding author on reasonable request.

## Abbreviations

BD: Biodentine
CSCs: Calcium silicate-based cements
σ_0_: Characteristic bond strength
C: Control
HF: Hydrofluoric acid
RP: Reblida post
RX: RelyX post
SB: Sandblasting
WMTA: White mineral trioxide aggregate
